# Basaloid carcinoma of the lung associated with central cavitation: a unique surgical case focusing on cytological and immunohistochemical findings

**DOI:** 10.1186/1746-1596-7-175

**Published:** 2012-12-11

**Authors:** Sohsuke Yamada, Hirotsugu Noguchi, Atsunori Nabeshima, Takashi Tasaki, Shohei Kitada, Tetsuro Baba, Hidetaka Uramoto, Takeshi Hanagiri, Yasuyuki Sasaguri

**Affiliations:** 1 Departments Of Pathology And Cell Biology, School Of Medicine, University Of Occupational And Environmental Health, Kitakyushu, Japan; 2 Departments Of Surgery II, School Of Medicine, University Of Occupational And Environmental Health, Kitakyushu, Japan

**Keywords:** Basaloid carcinoma, Lung, Cytology, Cavitation

## Abstract

A history of an increase in pulmonary mass was presented in the right upper lobe of a 72-year-old male. The bronchial brushing cytology specimens contained many sheet-like or three-dimensional clusters of malignant cells having small to medium-sized, uniform oval to round, and hyperchromatic nuclei, inconspicuous nucleoli, and scanty cytoplasm, admixed with mitotic figures. A coarsely granular chromatin pattern was predominantly noted. We first interpreted it as suspicious of malignancy, such as atypical carcinoid. A right upper lobectomy was performed, and gross examination revealed a centrally cavity-formed tumor lesion, containing asymmetrically thinned wall and looking grayish to whitish, partly adjacent to the bronchiolar wall. On microscopic examination, the tumor was predominantly composed of a solid proliferation of atypical epithelial cells without apparent glandular or squamous differentiation, often arranged in an alveolar growth pattern with peripheral palisading. Immunohistochemically, these atypical cells are negative for all three neuroendocrine markers and thyroid transcription factor 1, whereas positive for 34βE12, p63 and S-100 protein. Therefore, we finally made a diagnosis of basaloid carcinoma with cavity formation. We should be aware that, owing to its characteristic features, cytopathologists might be able to raise basaloid carcinoma of the lung as one of differential diagnoses, based on careful cytological examination.

**Virtual slides:**

The virtual slide(s) for this article can be found here: http://www.dianosticpathology.diagnomx.eu/vs/1519986488570234

## Background

Although basaloid carcinoma (BC) of the lung is a rare subtype of nonsmall cell lung cancer, one original paper described that up to 32% tumors were reclassified as BCs among a large series of surgically resected lung cancers with retrospective review, originally classified as poorly or undifferentiated carcinoma [1]. In 1975, Spencer first reported the histopathologic, immunohistochemical, ultrastructural, and clinical features of BC of the lung [2]. Subsequently, this entity was established based on the following criteria, as previously described by Brambilla *et al.* in 1992 [3]: *(1)* A solid lobular or anastomotic trabecular pattern growing invasively in a finger-like fashion from the bronchial and/or glandular duct lining; *(2)* Small cuboidal to fusiform cells of mean diameter 12 to 15 μm, with moderately hyperchromatic nuclei and without prominent nucleoli. There was a scant but visible cytoplasm, and no nuclear molding; *(3)* Peripheral palisading with radially arranged cells at the periphery of lobules; *(4)* A high rate of mitosis, between 15 and 44 per 10 high-power fields. The World Health Organization (WHO) classification of tumours of the lung now records carcinoma with basaloid pattern, either as a pure BC, a variant of large cell carcinoma with above typical histopathologic patterns, or as a basaloid variant of squamous cell carcinoma when coexisted with areas of squamous differentiation [4]. BC of the lung often poses a diagnostic challenge to clinicians and cytopathologists, since its entity is difficult to diagnose pre-operatively [5,6]. In fact, one old paper reported that the cytologic differentiation of BC extensively overlap with those of small cell carcinoma [5]. Although another group demonstrated that patients with BC of the lung did not have a poor prognosis than the other nonsmall cell lung cancers [7], Brambilla *et al.* recently have confirmed that lung carcinoma with a basaloid pattern is a unique entity with a significantly worse outcome [8], similar to BCs in organs other than the lungs [3]. Thus, it would be critical to establish an accurate preoperative diagnosis by bronchial brushing and washing cytology.

Indeed, pulmonary BC could be a relatively uncommon disease, but not as compared with some recently published case reports of extremely rare tumor cell types in the lung [9,10]. Despite of that, we report a unique surgical case of BC of the lung, associated with central cavitation. Based on the cytology specimens, we preoperatively interpreted it merely as suspicious of carcinoma.

## Materials and methods

The patient was a 72-year-old Japanese man. Bronchial brushing and washing cytology, and transbronchial lung biopsy from the pulmonary mass were performed, followed by a right upper lobectomy. The tumor specimens after fixation in 10% neutral buffered formalin were embedded in paraffin for histological or immunohistochemical examinations. All immunohistochemical stainings were carried out using Dako Envision kit (Dako Cytomation Co., Glostrup, Denmark) according to the manufacturer’s instructions, and using commercially available prediluted monoclonal antibodies against the following antigens: synaptophysin (Dako, diluted 1:20), chromogranin A (Dako, diluted 1:200), CD56 (NICHIREI, diluted 1:1), cytokeratins (34βE12; Leica Microsystems, Wetzlar, Germany, diluted 1:200, CK7; Dako, diluted 1:50, and CK20; Dako, diluted 1:60), p63 (Dako, diluted 1:30), S-100 protein (Dako, diluted 1:900), thyroid transcription factor 1 (TTF-1; Dako, diluted 1:100), α-smooth muscle actin (α-SMA; Dako, diluted 1:150), h-caldesmon (Dako, diluted 1:50), calponin (Dako, diluted 1:50), CD10 (NOVOCASTRA laboratories Ltd., Newcastle, United Kingdom, diluted 1:20), CEA (Dako, diluted 1:50), and Ki-67 (MIB-1; Dako, diluted 1:50). The profile of all these antigens is summarized in Table 1. Since all tumor specimens were fixed in formalin, transmission electron microscopy could not be performed.

**Table 1:** The profile of all immunohistochemical antigens

**Antigens**	**Sources of antibodies**	**Dilution**
34βE12	Leica Microsystems	diluted 1:200
CK7	Dako	diluted 1:50
CK20	Dako	diluted 1:60
Chromogranin A	Dako	diluted 1:200
Synaptophysin	Dako	diluted 1:20
CD56	NICHIREI	diluted 1:1
TTF-1	Dako	diluted 1:100
CEA	DAko	diluted 1:50
h-caldesmon	Dako	diluted 1:50
S-100 protein	Dako	diluted 1:900
p63	DAko	diluted 1:30
α-SMA	Dako	diluted 1:150
CD10	NOVOCASTRA	diluted 1:20
Calponin	Dako	diluted 1:50

## Case presentation

The patient had a history of cerebral infarction 10 years ago. He was a heavy smoker over 50 years. There was no history of malignancy, immunosuppressive disorders, use of immunosuppressive medications, or unusual infections.

During a follow-up of his infarction, a chest X-ray showed a mass shadow with central cavity area in the middle region of the right lung 1 and half years before the surgery. The sputum culture examined detected colonies of Mycobacterium Gordonae, however, following that, a recent increase of pulmonary mass was presented. Laboratory data, including blood cell count and chemistry, were almost within normal limits, except for high levels of blood urea nitrogen (BUN; 38 mg/dL) and creatinine (Cr; 1.87 mg/dL), manifesting as mild renal dysfunction. Carcinoembryonic antigen (CEA; 4.3 ng/mL), squamous cell carcinoma antigen (SCC; 5.3 ng/mL), cytokeratin 19 fragment (CYFRA; 5.3 ng/mL), neuron specific enolase (NSE; 13.5 ng/mL), and pro-gastrin-releasing peptide (pro-GRP; 78.8 pg/mL) levels as tumor markers were modestly increased up, but carbohydrate antigen (CA) 19–9 and sialyl Lewis X-i antigen (SLX) levels were within normal limits. A chest CT scan revealed a relatively well-demarcated mass, measuring approximately 37 x 30 x 23 mm, associated with central and variably thin-walled cavity formation, in the right upper lobe, S2. CT scans of the head and abdomen disclosed no definite evidence of metastases in the lymph nodes or other organs. The patient had neither recurrence nor metastases of the lung cancer, respectively, however, was dead due to bronchopneumonia at 3 years after the operation.

## Pathological findings

**Figure 1:**
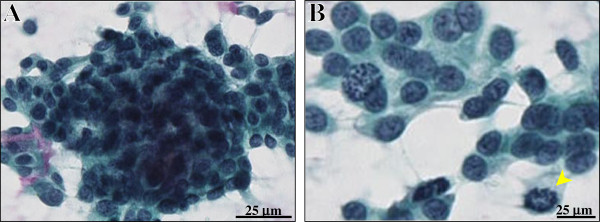
**Cytomorphologic examination of the bronchial brushing cytology specimens.** (**A**) The cytology specimens were consisted of many clusters of cohesive and sheet-like or three-dimensional tumor cells and a small number of individual tumor cells without necrotic or hemorrhagic backgrounds (Papanicolaou stains). Bar = 25 μm. (**B**) Those malignant cells showed small to medium-sized (12 to 20 μm in diameter), relatively uniform, and round to oval with mild pleomorphism and had relatively scanty cytoplasm. Additionally, the nuclei were hyperchromatic, predominantly in a coarsely granular chromatin pattern, and had inconspicuous nucleoli but mitotic figures (arrowhead) (Papanicolaou stains). Rosettes were absent. Bar = 25 μm.

The first bronchial brushing cytology specimens were consisted of many clusters of cohesive and sheet-like or three-dimensional tumor cells and a small number of individual tumor cells without necrotic or hemorrhagic backgrounds (Figure 1A). The bronchial washing cytology specimens were relatively inadequate, but very similar to the findings of brushing one. The malignant cells showed small to medium-sized (12 to 20 μm in diameter), relatively uniform, and round to oval with mild pleomorphism and had relatively scanty cytoplasm (Figure 1B). Additionally, the nuclei were hyperchromatic, predominantly in a coarsely granular chromatin pattern, and often had inconspicuous nucleoli, but occasionally mitotic figures (Figure 1B). Rosettes were absent, whereas a very small number of malignant squamoid cells was rarely seen. Based on that, we first interpreted it as suspicious of carcinoma, such as atypical carcinoid, and an ordinary right upper lobectomy was performed. On the other hand, the transbronchial lung biopsy specimens from the pulmonary mass were too small to be diagnostic.

**Figure 2:**
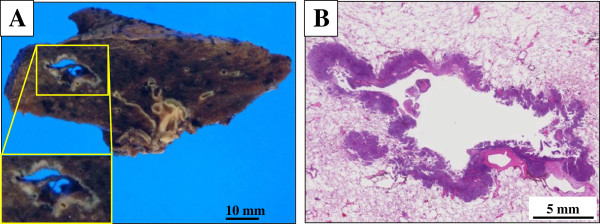
**Gross and microscopic examination of the resected specimen.** (**A**) On gross examination, the cut surface revealed a centrally cavity-formed, relatively poorly-demarcated, and solid firm mass, measuring 35 x 27 x 25 mm, which looked from grayish to whitish in color, partially adjacent to the bronchiole (lower side, inset). This central cavity measured approximately 30 x 10 mm, but filled with no necrobiotic materials. Bar = 10 mm. (**B**) A scanning magnification of it (H&E stains) showed that the cancer components were surrounded by the cavity and irregularly grew up along the asymmetrically thickened but relatively thin cavity wall, together with extension to the peripheral alveolar wall in a sheet-like fashion (lt. side). This tumor lesion was partly adjacent to the bronchiolo-vascular bundle (rt. lower side). There were no carcinoma *in situ* components in our case. Bar = 5 mm.

On gross examination, the cut surface revealed a centrally cavity-formed, relatively poorly-demarcated, and solid firm mass, measuring 35 x 27 x 25 mm, which looked from grayish to whitish in color, partially adjacent to the bronchiole (Figure 2A). This central cavity measured approximately 30 x 10 mm, but filled with no necrobiotic materials. The background of the lung had no remarkable change, i.e., not emphysematous (Figure 2A). A scanning magnification of it showed that the cancer components, less than 30% in volume, were surrounded by the cavity and irregularly grew up along the asymmetrically thickened but relatively thin cavity wall, together with extension to the peripheral alveolar wall in a sheet-like fashion (Figure 2B). This tumor lesion was partly adjacent to the bronchiolo-vascular bundle (Figure 2B). These features might indicate a sequential progression from the bronchiole to the surrounding alveolar space. There were no carcinoma *in situ* components within our thorough investigation.

**Figure 3:**
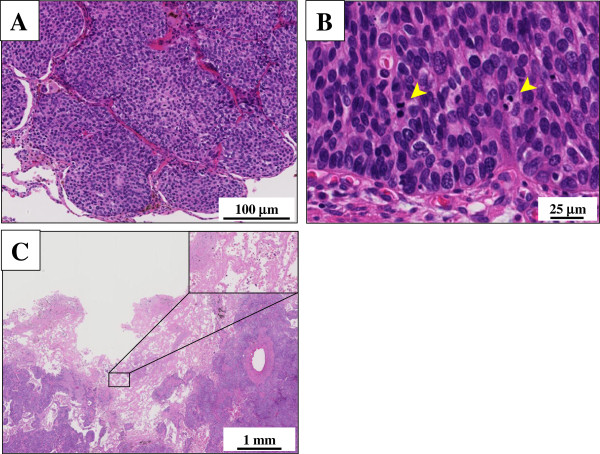
**Microscopic examination of the BC of the lung.** (**A**) Low power view showed a solid and sheet-like proliferation of relatively uniform and small to medium-sized atypical epithelial cells having hyperchromatic nuclei and scant eosinophilic cytoplasm, often arranged in an alveolar fashion with peripheral palisading. However, rosettes structures were absent. Apparent keratinization, intercellular bridge, or glandular differentiation was also not evident (H&E stains). Bar = 100 μm. (**B**) On high-power view, mitotic counts (arrowheads) were high (more than 15 per 2 mm^2^) (H&E stains). Bar = 25 μm. (**C**) Although foci of comedo-type tumor necrosis were not recognized within the cancer nests and the central cavity, the cancer-cavity junction contained coagulative necrosis of pre-existing alveolar wall (inset) (H&E stains). Bar = 1 mm.

**Figure 4:**
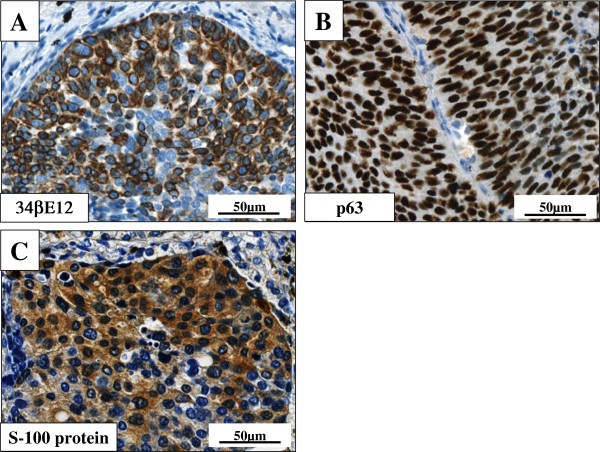
**Immunohistochemical examination of the BC of the lung.** (**A**, **B**, **C**) The carcinoma cells of BC were specifically positive for 34βE12 (A) and p63 (B). Additionally, one part of tumor nests was positive for S-100 protein (C). Bars = 50 μm.

Microscopic findings showed a solid and sheet-like proliferation of relatively uniform and small to medium-sized atypical epithelial cells having hyperchromatic nuclei and scant eosinophilic cytoplasm, often arranged in an alveolar fashion with peripheral palisading, typical of BC of the lung (Figure 3A). By contrast, rosettes structures were absent. Apparent keratinization, intercellular bridge, or glandular differentiation was also not evident, and there was not intracytoplasmic mucin with Alcian-Blue staining. On high-power view, mitotic counts were high (more than 15 per 2 mm^2^) (Figure 3B). The carcinoma cells partly involved the adjacent bronchiolar wall but without evidence of vessel permeation. Moreover, although foci of comedo-type tumor necrosis were not recognized within the cancer nests and the central cavity, the cancer-cavity junction sometimes contained coagulative necrosis of pre-existing alveolar wall (Figure 3C). Immunohistochemically, these carcinoma cells were negative for all three neuroendocrine markers, i.e., synaptophysin, chromogranin A and CD56, TTF-1, CEA, CK20, h-caldesmon, α-SMA, calponin, and CD10, but specifically positive for 34βE12 (Figure 4A), CK7 and p63 (Figure 4B). Additionally, one part of tumor nests was positive for S-100 protein (Figure 4C). On the other hand, MIB-1 labeling index was approximately 5% in the proliferating atypical cells of the cancer nests. All immunohistochemical profile of the carcinoma cells is summarized in Table 2.

**Table 2:** Immunohistochemical profile of the carcinoma components in our case of BC of the lung

**positive**	**negative**
34βE12	Chromogranin A
CK7	Synaptophysin
S-100 protein	CD56
p63	CK20
	TTF-1
	CEA
	h-caldesmon
	α-SMA
	CD10
	Calponin

Based on all these features, we suggested that these carcinoma cells were not characteristic of neuroendocrine, squamous, glandular, or transitional differentiation, and finally made a diagnosis of BC of the lung associated with central cavitation. Final pathological stage was determined as pT2aN0M0, stage IB, according to the International Association for Study of Lung Cancer (IASLC) classification [11].

## Discussion

Unlike the current BC case, frequent carcinoma *in situ* components should cause advanced clinical treatment, including more aggressive surgery or adjuvant chemotherapy, even in the early stage for BC of the lung [8]. It would lead to confer a significantly poor prognosis of BC amongst nonsmall cell lung cancer in stage I to II patients [8]. Thus, it could be critical to establish an accurate preoperative diagnosis by bronchial brushing and/or washing cytology, the clinical utility of which in diagnosing pulmonary tumors has been generalized. The cytological characteristic of BC of the lung would partly reflect the histopathological ones, showing cohesive, three-dimensional and/or sheet-like clusters of relatively small and uniform malignant cells, often having finely granular chromatin, inconspicuous nucleoli, high mitotic rate and scanty cytoplasm, arranged occasionally in a rosette-like pattern, as well as single cells formation in the background of possible necrosis [4-6]. However, in fact, the features of this relatively new and rare entity have not been well described or reviewed more recently. As in the present case, the cytology findings (Figure 1) showed almost similar to those as described above, even though neither rosette-like fashion nor necrotic backgrounds were evident. In spite of that, a confident and accurate diagnosis of BC of the lung might be impossible only on cytology specimens, likely due to lack of experience, cytomorphologic variety, misinterpretation and/or sampling errors. Nevertheless, in cases without evidence of neuroendocrine or squamous or glandular differentiation, such as ours, cytopathologists should raise possibility of BC as one of differential diagnoses, other than large cell neuroendocrine carcinoma, atypical carcinoid, small cell carcinoma, poorly differentiated squamous cell carcinoma or adenocarcinoma, or basaloid variant of squamous cell carcinoma, at the very least. Future studies will be further required after collecting and examining a larger number of pulmonary BC cases.

It is very likely that our case report is histopathologically remarkable for two reasons at least: first, central cavitation accompanied uniquely within the tissue of BC (Figure 2). Actually, to date, the number of ‘true’ cases reported as BC of the lung in the English literatures is not large, and most recent reference is from 2008 within our thorough investigation [8]. According to those papers, all BC tumors have exhibited nodular or mass lesions without cavity formation, not similar to our case. Furthermore, this case peculiarly showed coagulative necrotic foci of pre-existing alveolar wall in the cancer-cavity junction (Figure 3C), indicating ischemic change of the pulmonary tissue surrounded by the BC areas. Xue *et al.* have very recently proposed that the solitary thin-walled cavity of lung adenocarcinoma would be formed via multiple processes: adenocarcinoma cells initially develop in alveolar wall and grow toward bronchiole, and next formed a unidirectional check-valve owing to lack of cartilage in bronchiole; the accumulations of gases enter alveoli; the alveoli rupture and fuse into cavity with separation; and finally, the cavity gradually gets larger and larger with the increased inner pressure [12]. As in our BC case, it was suggested that the carcinoma cells firstly developed in the bronchiolar wall and subsequently grew extensively toward alveolar wall, and *vice versa*, since BC of the lung could originate from a basal bronchial or bronchiolar epithelial stem cell, as described by Brambilla *et al.*[3]. In this scenario, the above peculiar histopathological findings (Figure 3C) might confirm their above hypotheses with regard to the pathogenetic mechanisms of solitary thin-walled cavitation in lung cancer [12]. It would be intriguing to study this topic after investigating many cases of it. Second, immunohistochemical expression of not merely p63 but S-100 protein was positively seen in the tumor nests (Figure 4). Although there have been no large, detailed immunohistochemical studies of BC of the lung until now, the results indicate that those tumor cells have potential myoepithelial phenotypes, as well. We could provide the possible evidence for the first time that BC of the lung might arise from a ductal epithelial-myoepithelial cell, as a result of neoplastic transformation of outer supporting myoepithelial cells, as well as inner ductal epithelial cells [13]. However, since other myoepithelial markers examined, such as α-SMA, calponin, and CD10, were negative (Table 2), this suggestion may be highly speculative and partly unsupported. Despite of that, future convincing data will be further required to determine whether our hypothesis is significant or not.

## Conclusion

We herein reported a rare case of BC of the lung associated with central cavitation. The present case was tentatively diagnosed as suspicious of carcinoma, not otherwise specified, on the cytology specimens, since its features showed unclear differentiation. All cytopathologists should be aware that its cytomorphologically characteristic findings from extensively careful examination might induce one of differential diagnoses, and possibly a correct diagnosis. BC of the lung may be more common than generally considered.

## Consent

Written informed consent was obtained from the patient for publication of this case report and any accompanying images. A copy of the written consent is available for review by the Editor-in-Chief of this journal.

## Competing interests

The authors declare that they have no competing interests.

## Authors’ contributions

SY and HN participated in conception of the idea and writing of the manuscript. SY, HN, TT, AN, SK, TB, HU, TH and YS performed the cytohistological and immunohistochemical interpretation of the tumor tissue. All authors have read and approved the final manuscript.

## References

[B1] Moro D Brichon PY Brambilla E Veale D Labat F Brambilla C Basaloid bronchial carcinoma. A histologic group with a poor prognosis Cancer 1994 73 2374 2739 10.1002/1097-0142(19940601)73:11<2734::aid-cncr2820731114>3.0.co;2-4 8194014

[B2] Spencer H Pathology of the lung 1975 Oxford: Pergamon Press

[B3] Brambilla E Moro D Veale D Brichon PY Stoebner P Paramelle B Brambilla C Basal cell (basaloid) carcinoma of the lung: a new morphologic and phenotypic entity with separate prognostic significance Hum Pathol 1992 23 993 1003 10.1016/0046-8177(92)90260-A 1381335

[B4] Travis WD Colby TV Corrin B Shimosato Y Brambilla E Brambilla E, Pugatch B, Geisinger K, Gal A, Sheppard MN, Guinee DG, Jiang SX, Lantuejoul S, Chang YL, Peterson I, Meyerson M, Hanash SM, Noguchi M In collaboration with Sobin LH and pathologists from 14 countries: Large cell carcinoma Histological typing of lung and pleural tumours. World health organization. International histological classification of tumors 1999 Berlin: Springer 45 50

[B5] Dugan JM Cytologic diagnosis of basal cell (basaloid) carcinoma of the lung. A report of two cases Acta Cytol 1995 39 539 542 7762348

[B6] Foroulis CN Iliadis KH Mauroudis PM Kosmidis PA Basaloid carcinoma, a rare primary lung neoplasm: report of a case and review of the literature Lung Cancer 2002 35 335 338 10.1016/S0169-5002(01)00427-5 11844610

[B7] Kim DJ Kim KD Shin DH Ro JY Chung KY Basaloid carcinoma of the lung: a really dismal histologic variant? Ann Thorac Surg 2003 76 1833 1837 10.1016/S0003-4975(03)01296-7 14667594

[B8] Moro-Sibilot D Lantuejoul S Diab S Moulai N Aubert A Timsit JF Brambilla C Brichon PY Brambilla E Lung carcinomas with a basaloid pattern: a study of 90 cases focusing on their poor prognosis Eur Respir J 2008 31 854 859 10.1183/09031936.00058507 18094005

[B9] Bohn OL León EA Lezama O Rios-Luna NP Sánchez-Sosa S Llombart-Bosch A Pulmonary artery sarcoma with angiosarcoma phenotype mimicking pleomorphic malignant fibrous histiocytoma: a case report Diagn Pathol 2012 7 154 10.1186/1746-1596-7-154 23134683 PMC3538549

[B10] Gong L Liu XY Zhang WD Zhu SJ Yao L Han XJ Lan M Li YH Zhang W Primary pulmonary malignant melanoma: a clinicopathologic study of two cases Diagn Pathol 2012 7 123 10.1186/1746-1596-7-123 22992473 PMC3502413

[B11] Groome PA Bolejack V Crowley JJ Kennedy C Krasnik M Sobin LH Goldstraw P IASLC International Staging Committee; Cancer Research and Biostatistics; Observers to the Committee; Participating Institutions The IASLC lung cancer staging project: validation of the proposals for revision of the T, N, and M descriptors and consequent stage groupings in the forthcoming (seventh) edition of the TNM classification of malignant tumours J Thorac Oncol 2007 2 694 705 10.1097/JTO.0b013e31812d05d5 17762335

[B12] Xue X Wang P Xue Q Wang N Zhang L Sun J Wang K Yang B Wang J Comparative study of solitary thin-walled cavity lung cancer with computed tomography and pathological findings Lung Cancer 2012 78 45 50 10.1016/j.lungcan.2012.06.004 22784387

[B13] Yamada S Nabeshima A Tabata T Guo X Tasaki T Wang KY Shimajiri S Sasaguri Y Invasive salivary duct carcinoma ex pleomorphic adenoma of the parotid gland: a teaching case giving rise to the genuine diagnostic difficulty on an inadequate cytology specimen Diagn Pathol 2012 7 61 10.1186/1746-1596-7-61 22647549 PMC3497703

